# Inbreeding Depression and Purging in Fertility and Longevity Traits in Sheep Breeds from Germany

**DOI:** 10.3390/ani14223214

**Published:** 2024-11-08

**Authors:** Cathrin Justinski, Jens Wilkens, Ottmar Distl

**Affiliations:** 1 Institute of Animal Breeding and Genetics, University of Veterinary Medicine Hannover (Foundation), 30559 Hannover, Germany; cathrin.justinski@tiho-hannover.de; 2 VIT—Vereinigte Informationssysteme Tierhaltung w.V., Heinrich-Schröder-Weg 1, 27283 Verden, Germany; jens.wilkens@vit.de

**Keywords:** ancestral inbreeding, new inbreeding, heritability, number of lambings, animal model, lambs born, lifetime, productive life

## Abstract

In the present study, we analysed fertility and longevity traits of 22 sheep breeds from Germany with a suitable quantity of data in the national database OviCap. The data comprised merino, meat, country and milk sheep breeds with 62,198 ewes and about 173,000 lambing records, until the fifth lambing. Across-breed means of heritabilities reached estimates of 0.13, 0.17 and 0.18 for number of lambings, average number of lambs born per lambing and number of lambs per lifetime, respectively. For age at first lambing, length of lifetime and productive life, mean heritabilities over breeds were 0.34, 0.17 and 0.32, respectively. The across-breed means of the individual rate of inbreeding were significantly negative for the average number of lambs born per lambing and number of lambs born per lifetime, and for number of lambings it was close to the significance threshold. We found declining slopes for inbreeding depression for the average number of lambs born per lambing and number of lambs born per lifetime in 16 breeds, and significantly negative slopes in five and seven breeds. For lifetime and productive life, 9/22 and 8/22 breeds showed significant inbreeding depression, while for age at first lambing, only 1/22 breeds showed significant inbreeding depression. A significant reduction in inbreeding depression due to purging effects was found for eight breeds. Fitness traits may be subject to forced directional selection. Therefore, sheep breeding programmes should give special consideration to fertility and longevity traits. Fitness related traits seem to be essential in conservation of genetic diversity within sheep breeds.

## Simple Summary

The objectives of the present study were to analyse genetic parameters and the effects of inbreeding on the average number of lambs born per lambing, the age at first lambing, the number of lambings, the number of lambs born in the lifetime and the survival to the fifth lambing using length of lifetime and productive life. The study comprised 62,198 ewes of 22 sheep breeds in Germany with about 173,000 registered lambings. Overall-breed means for heritabilities ranged from 0.17–0.18 for traits including number lambs born and from 0.17–0.34 for longevity traits. The across-breed averages for inbreeding depression, expressed by the individual rate of inbreeding, were significantly negative for the average number of lambs born per lambing and number of lambs born per lifetime, and for number of lambings it was close to the significance threshold. Slopes for inbreeding depression for the latter traits were negative for 14–16 breeds, and significantly negative for 5/22, 7/22 and 8/22 breeds, respectively. A significant reduction of inbreeding depression due to purging was found for eight breeds. Therefore, fitness traits may be subject to forced directional selection. Fertility and longevity traits must be given special consideration in sheep breeding to maintain genetic diversity within breeds.

## 1. Introduction

In recent decades, researchers’ interest in the genetics of animal health has been growing. In particular, the effects of inbreeding depression on the fertility and health of sheep populations are of great importance [1,2]. The Merino sheep, a common breed of sheep, provides an interesting case study to further investigate the relationships between inbreeding depression and fertility [3]. Many studies have already demonstrated the effects of inbreeding in numerous selection traits of different sheep breeds and have shown that inbreeding depression can be manifold in breeding populations [1,4,5]. In addition to other production traits, fertility is a decisive factor for the profitability of sheep breeding [6], as it has a direct impact on the reproduction rate and breeding success [7]. Fertility traits include number of lambs born and born alive, the individual birth weight, birth type (singleton, multiple), etc. and concern both the lambs and the dams [8]. Analla et al. investigated the effects of litter size and heterosis and showed that multiple lambs are less subject to strong influences [9,10]. A similar study was performed for Turkish Karacabey Merino [11]. It appears that the use of inbreeding as a means of improving productive performance in sheep is more likely to be a disadvantage, economically and genetically, than an advantage. This finding is demonstrated by a comprehensive study of the effects of inbreeding on reproductive traits in three sheep breeds related to Merino sheep [12]. Gowane et al., on the other hand, found no significant inbreeding effects on any of the traits analysed in Bharat Merino [13]. Beni Guil lambs were found to have inbreeding depression in both birth weight and 30- and 90-day weight [14]. In the Hampshire sheep, inbreeding had impact on several fertility traits [15]. In three of the Danish meat sheep breeds, inbreeding depression was found for all traits. For most combinations of traits and breeds, there was also a significant reduction in phenotype values due to inbreeding in the dam [16]. For litter size, which Vostry et al. analysed in Romanov sheep, inbreeding effects on litter size were statistically significant, but very small (around −0.05 lambs per 1% inbreeding change) [17]. Murphy et al. estimated heritabilities for various reproduction-associated traits in Romanov sheep [18]. Selvaggi et al. found inbreeding effects on birth weights in the Italian Leccese sheep and all other reproductive traits analysed in this study also showed strong decreases in connection with an increasing inbreeding rate [7]. In the Elsenburg Dormer sheep, estimates of inbreeding depression were −0.006 kg for birth weight and −0.093 kg for weaning weight per 1% increase of individual inbreeding [19].

In this study, we focus on sheep breeds from Germany and investigate the magnitude to which inbreeding depression and purging affect fertility and longevity traits. Since sheep populations have small effective population sizes, inbreeding cannot be avoided [1,2,3,4,5,6,7,8,9,10,11,12,13,14,15,16,17,18,19,20]. Inbreeding and inbreeding depression in production traits have already been shown for sheep breeds from Germany [4,5,20]. Traits subject to forced selection and fitness-related traits are particularly sensitive to inbreeding depression. The magnitude depends on the degree to which recessive alleles will be harmful to fitness, when homozygous, and if loss of heterozygosity also leads to reduced fitness [1]. In addition, we analysed the genetic parameters for these traits that were employed to estimate linear regression slopes for inbreeding coefficients. The outcomes of the present analyses should supply important and novel insights for the sheep sector and contribute to the development of breeding strategies to improve the longevity and fertility of sheep breeds [21,22]. To ensure efficient sheep production in the future, a high level of prolificacy in sheep has to be maintained. Inbreeding depression can result in reduced levels of prolificacy in sheep populations, particularly when population sizes are small and selection is performed on several trait complexes.

The objectives of the present work were therefore to analyse the effects of inbreeding on fertility and longevity in all sheep breeds in Germany that are recorded for fertility parameters as part of their breeding programmes. We employed linear regression coefficients in animal models to estimate inbreeding depression and purging effects. In addition, we compared overall mean values of inbreeding effects for fertility and longevity traits with those of the production traits in sheep breeds from Germany [4,5] and results of previous studies [1,2,3,6,7,8,9,10,11,12,13,14,15,16,17,18,19].

## 2. Materials and Methods

### 2.1. Data Editing and Selection of Sheep Breeds

The nationwide database OviCap for sheep and goats in vit/Verden, Germany, was used to retrieve data recorded for reproduction as well as birth and culling dates. Users of the database OviCap can also retrieve breed characteristics, breeding objectives, breeding programmes and images for all sheep breeds registered in a German herdbook (https://service.vit.de/dateien/ovicap/musterzuchtprogramme.html, accessed on 30 October 2024). Breeds considered for this study had more than 200–500 animals with consecutive records for lambing data in the years 2010–2022, as well as sufficient pedigree records. Restrictions on pedigree records are defined through complete equivalent generations (GEs) with a threshold of GE > 3.5. Therefore, not all breeds used in our previous work [20] could be included in the present analysis. Finally, 22 sheep breeds had sufficient reproduction, lifetime and pedigree records to be selected for the present study. Reproductive traits, birth and culling dates are mandatory for all female herdbook animals.

In German herdbook sheep breeds, for each lambing the date of lambing, number of lambs born, sex of the lambs, dam and sire of the lambs have to be recorded by the farmer via the lambing report and these data are transferred to OviCap. Data are tested for plausibility by vit/Verden and have to be corrected by the animal owner in cases of inconsistency. Each reported lambing date is compared with the birth date of the dam and sire as well with the current date and the preceding lambing date for the second and subsequent lambings. Dates seeming not plausible had to be corrected by the animal owner.

We edited the data for the lambing years 2010–2022. Only ewes were included which were born before 2015 and had the first five lambings until 2022 or left the flock in or before 2022 (Supplementary Materials Table S1). This way we were able to collate for each ewe a minimum number of five lambings without censoring of the data. For the analysis of reproduction and longevity traits, we thus restricted these traits to a maximum of five lambings.

### 2.2. Traits Analysed

The traits in this study comprised six different traits for reproduction or longevity. We calculated the number of lambings (Lambings), the average number of lambs born per lambing (Lambs-S), the number of lambs born per lifetime in years (Lambs-L), age at the first lambing (AFL), length of lifetime in years (Lifetime) and length of productive life in years (Prod-Life). Average values with their standard deviations were 2.789 ± 1.432 (Lambings), 1.521 ± 0.460 (Lambs-S), 1.081 ± 0.447 (Lambs-L), 741 ± 222 (AFL), 3.845 ± 1.527 (Lifetime) and 1.814 ± 1.479 (Prod-Life). Mean values, standard deviations, minima and maxima by breed are given in Supplementary Materials Table S2.

The number of lambings included the first five lambings or less, when the ewe left the flock before the fifth lambing. Therefore, a ewe could have 1–5 lambings in the edited data set. Analogously, the average number of lambs born per lambing and number of lambs born per lifetime in years were restricted to the first five lambings. Ewes with less than five lambings and a culling date were regarded with the respective lambings recorded. Lifetime was defined as the time-period from birth to fifth lambing or to the date when the ewe left the flock before the fifth lambing. Correspondingly, productive life was from the first lambing until to the fifth lambing or to the date when the ewe left the flock before the fifth lambing.

### 2.3. Statistical Analysis

The inbreeding coefficients according to Meuwissen and Luo [23], Ballou [24], Kalinowski et al. [25] and Baumung et al. [26] calculated in our previous study [20] using PEDIG [27] and GRAIN (version 2.2) [28] were used for this study.

The heritabilities, residual and genetic correlations and (co-)variances for the six reproductive traits including number of lambings, average number of lambs born per lambing, number of lambs per lifetime, age at the first lambing, lifetime from the birth to the fifth lambing and length of productive life from the first to the fifth lambing were estimated for each sheep breed.

The following linear multivariate animal models were used to estimate the variance and covariance components, which were parameterized according to the models used in the routine breeding value estimation of vit/Verden.

Model 1 for the traits number of lambings, average number of lambs born per lambing and number of lambs per lifetime was as follows:

Y_ijkl_ = µ + LOC-YEAR_i_ + bAGE_j_ + SEA_k_ + animal_l_ + e_ijkl_, (1)

where Y_ijkl_ = number of lambings or the average number of lambs born per lambing or the number of lambs per lifetime. LOC-YEAR_i_ is the ith flock by birth year with different numbers of levels for each breed; AGE_j_ = age at first lambing as a linear covariate with b as linear regression coefficient; SEA_k_ = lambing season in classes, k = 1 (December to May) or 2 (June to November); animal_l_ = random animal effect of the l-th animal; and e_ijkl_ = random residual error.

Model 2 for the traits age at the first lambing, length of lifetime in years and length of productive life in years was as follows:

Y_ijk_ = µ + LOC-YEAR_i_ + BMO_j_ + animal_k_ + e_ijk_, (2)

where Y_ijk_ = age at the first lambing or lifetime or productive life. LOC-YEAR_i_ is the fixed effect of the ith flock by birth year with different numbers of levels for each breed; BMO_j_ is the fixed effect of the month of birth, with j = 1–12; animal_k_ is the random additive genetic effect of the k-th animal; and e_ijk_ = random residual error.

The various inbreeding coefficients were analysed by adding linear regression coefficients (b_1_ to b_5_) to model 1 and 2 for the respective traits. We parameterized models for the individual rate of inbreeding (ΔF_i_) [29], the inbreeding coefficients according to Kalinowski et al. (2000) [25] and the classical inbreeding coefficient (F) [23] and its interaction with the inbreeding coefficient according to Ballou (1997) [24].

Model 3 considered the individual rate of inbreeding (ΔF_i_) [29] as linear regression.

Y_ijklm_ = µ + LOC-YEAR_i_ + bAGE_j_ + SEA_k_ + b_1_ΔF_il_ + animal_m_ + e_ijklm_

and

Y_ijkl_ = µ + LOC-YEAR_i_ + BMO_j_ + b_1_ΔF_ik_ + animal_l_ + e_ijkl_

Model 4, with the linear regressions of the ancestral inbreeding coefficient (F_a_Kal_) and the new inbreeding coefficient (F_a_New_), was applied according to Kalinowski et al. (2000) [25].

Y_ijklmn_ = LOC-YEAR_i_ + bAGE_j_ + SEA_k_ + b_2_F_a_Newl_ + b_3_F_a_Kalm_ + animal_n_ + e_ijklmn_

and

Y_ijklm_ = LOC-YEAR_i_ + BMO_j_ + b_2_F_a_Newk_ + b_3_F_a_Kall_ + animal_m_ + e_ijklm_

Model 5, with the linear regressions of the classical inbreeding coefficient (F) and the interaction of F_a_Bal_ with F, was applied as proposed by Ballou (1997) [24].

Y_ijklmn_ = LOC-YEAR_i_ + bAGE_j_ + SAE_k_ + b_4_F_l_ + b_5_F×F_a_Balm_ + animal_n_ + e_ijklmn_

and

Y_ijklm_ = LOC-YEAR_i_ + BMO_j_ + b_4_F_k_ + b_5_F×F_a_Ball_ + animal_m_ + e_ijklm_

The variance components were simultaneously estimated for the respective three traits using models 1 and 2 and VCE 6.0.2 [30]. We employed REML (restricted maximum likelihood) using analytical gradients for estimation of the variance and covariance components. For models 3–5, we used estimated additive genetic and residual variances to estimate effects for linear regressions using PEST, version 4.2.6. Further statistical analyses were performed using SAS, version 9.4 (Statistical Analysis System, Cary, NC, USA, 2023).

## 3. Results

### 3.1. Trait Means, Estimated Heritabilities, Residual and Genetic Correlations and (Co-)Variances

In this study, we investigated the genetic parameters of fertility and longevity traits of 22 sheep breeds (Table 1, Table 2 and Table 3, Supplementary Materials Tables S3 and S4). The number of complete equivalent generations (GEs), the number of animals with data records, the number of lambings and the heritability estimates with their standard errors are shown in Table 1. Heritabilities of the mean number of lambings ranged from 0.02 (BDC) to 0.54 (CHA). The mean (median) heritability for number of lambings was 0.13 (0.11) across all breeds. The values of the first and third quartile were 0.05 and 0.17 and included 12/22 breeds. The breeds CHA and WAD showed the highest estimates with 0.54 ± 0.08 and 0.25 ± 0.30, respectively, whereas BDC and MFS the lowest estimates with 0.02 ± 0.52 and 0.04 ± 0.02. High standard errors for heritability estimates were obtained for the breeds BDC, WAD, SWS and MLW.

The average number of lambs born per lambing and per lifetime with their heritabilities and standard errors are given in Table 2. For these traits, heritabilities were in the range from 0.05 (SWS) to 0.70 (BCD) and from 0.02 (SWS) to 0.53 (BCD). Means (medians) for these two traits were 0.17 (0.12) and 0.18 (0.16). The first and third quartiles were from 0.07 to 0.23 and from 0.11 to 0.22. The quartiles comprised 11/22 and 12/22 breeds.

Age at the first lambing, lifetime from the birth to the fifth lambing and length of productive life from the first to the fifth lambing showed mean (median) heritabilities of 0.34 (0.30), 0.17 (0.10) and 0.32 (0.29), respectively (Table 3). The highest estimates for these traits were obtained for COF (0.99 ± 0.01), CHA (0.97 ± 0.01) and COF (0.78 ± 0.03), respectively. Heritability estimates were lowest for MFS (0.08 ± 0.02), MLW (0.02 ± 0.01) and DOS (0.09 ± 0.02), respectively. The first and third quartiles of the three traits were 0.22–0.42, 0.06–0.14 and 0.22–0.42, respectively. The quartiles included 13 breeds for age at the first lambing and 12 breeds for the other two traits.

### 3.2. Inbreeding Depression and Purging

#### 3.2.1. Individual Rate of Inbreeding

Across breeds, we found significantly negative regression coefficients for the individual rate of inbreeding on the average number of lambs born per lambing and number of lambs per lifetime (Table 4). Across-breed means of the individual rate of inbreeding were negative for all traits, except AFL. In the sheep breeds, CHA, GGH, LES, SKU, SUF and WHH, an increase of the individual rate of inbreeding reduced number of lambings, number of lambs born, lifetime and productive life and prolonged age at first lambing (Table 5). For OMS and SKU, the linear regression coefficients were significantly negative for all traits, except AFL. Means of the linear regression coefficients for the individual rate of inbreeding were not significantly different between breeding directions (Supplementary Materials Tables S5–S10).

#### 3.2.2. Ancestral and New Inbreeding According to Kalinowski

The linear regression coefficients F_a_Kal_ and F_a_New_ were not significant for any trait analysed across breeds (Table 4). For number of lambs per lifetime, F_a_Kal_ was positive and F_a_New_ negative. For all other traits, the signs for both inbreeding coefficients were either positive (AFL) or negative. For the sheep breeds, DOS, OMS and SKF, and the trait number of lambings, F_a_Kal_ was significantly positive, whereas F_a_New_ was significantly negative (Supplementary Materials Tables S5–S10). Average number of lambs born per lambing for WHH showed a significantly positive F_a_Kal_, but a significantly negative F_a_New_. The same patterns for F_a_Kal_ and F_a_New_ in the signs and significance were found for the trait number of lambs per lifetime in the sheep breeds DOS and SKF. In addition, the same signs and significance for F_a_Kal_ and F_a_New_ were obtained for the trait length of lifetime in the sheep breeds MFS, MLW, OMS and SKF, as well as for the trait productive life in the sheep breeds MLW, OMS and SKF (Supplementary Materials Tables S5–S10). For the means of the linear regression coefficients F_a_Kal_ and F_a_New_, no significant differences could be found between breeding directions (Supplementary Materials Tables S5–S10).

#### 3.2.3. F and Interaction of F×F_a_Bal_

The linear regression coefficients of F and F×F_a_Bal_ across breeds were negative for all traits, except AFL (Table 4). Significance was only achieved for F by the average number of lambs born per lambing. For the breeds, GGH, MLW, OMS, SKF and WAD, F was significantly negative and F×F_a_Bal_ was significantly positive for the number of lambings. For the traits lifetime and productive life, significantly negative regressions for F and significantly positive regressions for F×F_a_Bal_ could be demonstrated for the breeds GGH, MFS, MLW, OMS and SKF. Means of the linear regression coefficients F and F×F_a_Bal_ did not significantly differ between breeding directions (Supplementary Materials Tables S5–S10).

### 3.3. Inbreeding Effects Relative to the Phenotypic Mean and Phenotypic and Additive Genetic Standard Deviation

Scaling the individual rates of inbreeding to the phenotypic mean and phenotypic and additive genetic standard deviation showed for the traits average number of lambs born per lambing and number of lambs per lifetime across breeds values which were significantly different from zero (Table 6). The across-breed mean (median) for AFL increases per 1% increase in ΔF_i_ scaled to the trait mean by 0.1372 (0.1877) and for the other traits a decrease by −0.1456 (−0.0519) to −0.6058 (−0.7151). Across-breed means for ΔF_i_/σ_P_ and ΔF_i_/σ_A_ were lowest for lifetime and second lowest for number of lambings.

## 4. Discussion

Our results revealed a significant decrease of the overall breed mean of the average number of lambs born per lambing and the number of lambs per lifetime by −0.4142% and −0.6058%, as a 1% increase in individual inbreeding rate by phenotypic trait mean. In terms per phenotypic and additive genetic standard deviations, the 1% increase of inbreeding depression across breeds reached 1.7037% and 2.2870% of the phenotypic standard deviation and 5.3126% and 7.8731 of the additive genetic standard deviation. For these two latter traits, 16/22 sheep breeds showed negative regression slopes for the individual rate of inbreeding. The six sheep breeds MLS, MLW, OMS, SKU, SUF and SWS, in particular, showed significant and steeply declining slopes. Inbreeding depression was significant for longevity traits (number of lambings, lifetime, productive life) in more sheep breeds significant (8–9 breeds) than for traits of litter size (5 and 7 breeds). However, across-breed means were not significant for longevity traits because the inbreeding effects were also positive for a larger number of breeds (negative for 8–12 breeds versus positive for 6 breeds) in these traits.

A linear decline in the values of fertility and longevity traits with increasing levels of individual inbreeding rate occurs when dominance effects influence these traits [1,31,32]. With partial dominance of loci affecting the trait, an increasing frequency of deleterious recessive alleles in homozygous genotypes with negative effects on fitness traits is associated with inbreeding depression [1,31,32]. Another explanation could be the frequency of favourable heterozygous genotypes with overdominance, which confer higher fertility and longevity [31,33]. As inbreeding reduces the frequency of heterozygotes, the beneficial effects of heterozygous loci are lost, leading to inbreeding depression. Both hypotheses seem to be competing [1,31,32,33], but we cannot exclude the possibility that the effects of deleterious alleles differ in magnitude [31,32,34,35] and add up to negative inbreeding effects across all loci. This could also be the case in the present study, where we found inbreeding depression in fitness-related traits for 10–16 out of 22 breeds, but significant decreases in slopes were only found in 5–9 breeds. These differences between breeds and traits could be related to the magnitude of the effects of the deleterious alleles, whereas overdominance may play a minor role in inbreeding depression [1,31,32,33]. Another hypothesis based on partial inbreeding coefficients could also help to explain the differences in inbreeding depression between sheep breeds. Inbreeding depression differed significantly between major founders in several studies [31]. This result suggests the possibility that differences in the frequency of damaging founder alleles may influence the extent of inbreeding depression. Follow-up analyses can test this hypothesis, especially with regard to conservation strategies for endangered sheep breeds. The role of epistatic effects on inbreeding depression is generally thought to be small, as linear models fit the data well when inbreeding coefficients are <10–20% [36,37,38]. Therefore, we did not use nonlinear regressions on inbreeding coefficients [4,5].

In all sheep breeds, fertility is the most economically important trait, and therefore this trait is under selection pressure in all sheep breeds and consequently under an increasing risk of inbreeding depression [1,2,3,4,5,6,7,8,9,10,11,12,13,14,15,16,17,18,19,31,32]. Lifetime and productive life depend on fertility and therefore longevity traits may be assumed to be exposed to a similar forced selection as fertility traits. In the breeds BLS, COF, MLS, RPL, SWS and TEX, significant positive effects were observed despite of an increasing rate of individual inbreeding. In MLS, purging effects seem to act as indicated by significant positive ancestral inbreeding F_a_Kal_ and F×F_a_Bal_ effects and significant negative effects due to new inbreeding and F. While for the breeds BLS, COF, RPL and SWS, F_a_New_ exhibited significant positive effects and thus may have contributed to the positive slopes with increasing individual rates of inbreeding. In the breed TEX, both regression coefficients for F_a_New_ and F_a_Kal_ were significantly positive, but the F×F_a_Bal_ effects were significantly negative. This may indicate that increasing individual rates of inbreeding in the TEX breed had not so great effects and the accumulation of inbreeding was only so great for F_a_Bal_ that in most of the animals with more strongly inbred ancestors negative effects on longevity became evident.

Indications for purging were evident for the sheep breeds MFS, MLW, OMS and SKF when analysing the slopes for ancestral and new inbreeding, as well as for F and F×F_a_Bal_ on longevity traits. For the breed DOS, results for F_a_New_ and F_a_Kal_ were indicative for purging on longevity, while for the breeds GGH and WAD, results for F and F×F_a_Bal_ were significant. In addition, for the breed WHH, the slopes for F_a_New_ and F_a_Kal_ indicated purging on the average number of lambs born per lambing. In comparison to previous reports, this is the first time that purging effects appeared likely in sheep. The breeds OMS and MLW in particular appear to be very susceptible to inbreeding depression as shown by the significant negative slopes for the increase in the individual rate of inbreeding in fertility and longevity traits (5/6 and 3/6 traits affected), whereas this did not appear to be the case for the breeds MFS and SKF, as these slopes were rather flat. Therefore, the results of our study support the hypothesis that in sheep breeds, traits that are intensively selected are more prone to inbreeding depression [1,2,3,4,5,6,7,8,9,10,11,12,13,14,15,16,17,18,19,31,32]. At the same time in these breeds, purging effects may also become visible [31,32,39,40,41,42]. Increased inbreeding may have significant detrimental effects on fertility and longevity traits in the OMS and MLW breeds in particular. Due to the great economic importance for sheep production, reduced fertility, infertility and health problems may cause involuntary culling of ewes and this way natural selection may contribute to purging.

Purging the genetic load may only occur when deleterious alleles with large effects are responsible for inbreeding depression and the population is under strong selection pressure [1,31,32,39,40,41,42]. Therefore, we propose that alleles detrimental for longevity traits were accumulated through continued mating among relatives in the sheep breeds MFS, MLW, OMS and SKF, in particular, and even in the breeds DOS and GGH. In addition, we may also assume that the effects of these alleles, when homozygous, were moderate to large. In MLW, OMS, GGH and WAD, deleterious alleles with small to large effects may be present because purging did not eliminate the genetic load resulting from less deleterious alleles. Therefore, we found strong inbreeding depression in MLW, OMS, GGH and WAD and at the same time significant purging. Furthermore, loss of heterozygosity could have also contributed to the magnitude of inbreeding depression. In contrast, in DOS, MFS and SKF, purging seemed to have effectively reduced the genetic load as the inbreeding depression was no longer significant and the regression slopes were close to zero or positive.

In summary, the present data and previous data [1,4,5,31,32,33,34,35,43] let us propose that traits under forced selection in German sheep breeds are affected significantly more often by inbreeding depression. These traits include wool quality, muscle conformation, exterior, fertility, number of lambings, length of lifetime and productive life. These traits may be expected to be under combined directional selection pressure and be influenced by favourable dominance effects. In this case, inbreeding depression is expected, considering the relationship between inbreeding depression and favourable dominance effects (=d_i_) over loci with $-F\sum_{i}2p_{i}q_{i}d_{i}$ [1,31,32]. Particularly, fertility and longevity traits are related to fitness and therefore positive dominance effects are very likely [1,31,32].

The present results allow comparisons of the inbreeding depression between the fitness-related traits with conformation and production traits for all German sheep breeds in the herdbook with a sufficient amount of data [4,5]. Comparing the across breed medians of the inbreeding depression relative to the phenotypic standard deviation per 1% increase in ΔF_i_ of the fertility and longevity traits with the corresponding estimates for wool quality, muscle conformation and exterior score, shows that the conformation traits with estimates of −1.29% (wool quality and muscle conformation) and −1.25% (exterior) are in a similar range. Mean number of lambings and number of lambs per lifetime resulted in lower estimates with −1.8584% and −2.5742%, respectively, while the other traits did not reach such low estimates with −1.1294% (average number of lambs born per lambing), −0.8084% (productive life), −0.7392% (lifetime) and 0.6198% (age at first lambing). Similar results can be seen when compared with meat performance, such as daily weight gain (−2.3955%), meatiness score (−1.2395%), ultrasound muscle (−1.9737%) and fat thickness (0.1399%). These results support the hypothesis of a similar degree of inbreeding depression for the different categories of traits across German sheep breeds [1,4,5,31]. This result may also reflect that selection intensities for the different trait complexes may be similar and similar effect sizes of the alleles with negative effects on the different trait complexes, when comparisons are across all sheep breeds.

The meta-analysis of Doekes et al. [1] showed an overall median and a median for the trait category reproduction for inbreeding depression of −0.52% and −0.38%, for the trait category reproduction, evaluated in terms of a 1% increase in F and calibrated to the phenotypic standard deviation. The estimates of our study for all fertility and longevity traits indicated a greater inbreeding depression with an overall estimate of −0.482% when compared to the reproductive traits of Doekes et al. [1] (Supplementary Materials Table S11). When leaving out the trait AFL from the present study, we obtained an overall estimate of −0.578%, which even indicated a higher inbreeding depression in German sheep breeds in the traits studied here than the overall median of −0.52% from Doekes et al. [1]. The overall median for conformation, meat, fertility and longevity of the German sheep breeds resulted in an estimate of −0.43% or −0.46% (without AFL), which indicates less inbreeding depression than the overall median of Doekes et al. [1].

Breeding programmes should take into consideration the intensity of selection for production- and fitness-related traits, the development of inbreeding and their effects on breeding objective traits in order to keep inbreeding depression as low as possible. The results of the present study should be useful in optimizing breeding programmes, particularly for threatened sheep breeds, to balance selection pressure on production and fitness traits and measures for maintaining genetic diversity in German sheep breeds.

## 5. Conclusions

The results of this study demonstrated significant inbreeding depression for fertility and longevity traits in 5/22 to 9/22 breeds. Across-breed means and medians were significant for average number of lambs born per lambing and number of lambs per lifetime. Inbreeding depression appeared as most important for number of lambings and number of lambs per lifetime, when presented in medians as a 1% increase in ΔF_i_ and calibrated to the phenotypic and additive genetic standard deviation. For the breeds MFS, MLW, OMS and SKF, we could demonstrate significant purging in all longevity traits, and for the breed WHH, significant purging for average number lambs born per lambing. Purging the inbreeding load in MFS and SKF was successful in reducing the inbreeding depression for longevity. In contrary, in MLW and OMS inbreeding depression persists despite the reduction of the genetic load. Summarizing the previous and present results, we found, per 1% increase in ΔF_i_, an overall median decrease of the trait values by −1.606% and −4.213% in units of the phenotypic and additive genetic standard deviations.

## Figures and Tables

**Table 1:** Survey on sheep breeds studied with their breeding directions (BD) and complete equivalent generations (GEs), number of animals per breed (N), trait values (means and standard deviations) for the number of lambings and the heritabilities (h^2^) with their standard errors.

Code	Breed	BD	GE	Number of Lambings		
				N	X ± SD	h^2^
BDC	Berrichon du Cher	EXO	3.67	306	2.63 ± 1.37	0.02 ± 0.52
BLS	Bentheim	CON	8.04	2153	3.03 ± 1.45	0.21 ± 0.05
CHA	Charollais	MEA	3.21	1149	2.34 ± 1.29	0.54 ± 0.08
COF	Coburg	CON	7.62	2647	2.75 ± 1.42	0.08 ± 0.05
DOS	Dorper	MEA	5.82	1899	2.82 ± 1.44	0.07 ± 0.03
GGH	German Grey Heath	HEA	7.52	3255	2.81 ± 1.46	0.05 ± 0.03
IDF	Ile-de-France	MEA	3.73	605	2.90 ± 1.50	0.06 ± 0.03
LES	Leine	CON	7.61	2601	2.93 ± 1.44	0.14 ± 0.04
MFS	German Mutton Merino	MER	6.68	4815	3.02 ± 1.41	0.04 ± 0.02
MLS	German Merino	MER	8.42	9406	2.92 ± 1.45	0.15 ± 0.02
MLW	Merino Longwool	MER	6.59	3166	2.95 ± 1.41	0.05 ± 0.14
OMS	East Friesian	MIL	8.20	3178	2.60 ± 1.40	0.12 ± 0.05
RPL	Pomeranian Coarsewool	CON	7.47	3023	2.67 ± 1.39	0.19 ± 0.04
SKF	German Blackhead Mutton	MEA	7.80	6885	2.92 ± 1.44	0.05 ± 0.01
SKU	Skudde	HEA	6.50	1747	2.27 ± 1.30	0.17 ± 0.06
SUF	Suffolk	MEA	5.25	4438	2.65 ± 1.42	0.12 ± 0.03
SWS	Swifter	MEA	3.71	251	2.82 ± 1.47	0.21 ± 0.15
TEX	Texel	MEA	5.71	4397	2.62 ± 1.37	0.04 ± 0.03
WAD	Wald	CON	5.65	559	2.55 ± 1.47	0.25 ± 0.30
WGH	German White Heath	HEA	7.37	1140	2.73 ± 1.43	0.17 ± 0.01
WHH	White Polled Heath	HEA	8.69	2982	2.88 ± 1.39	0.13 ± 0.04
WKF	German Whitehead Mutton	MEA	7.52	1596	2.77 ± 1.38	0.10 ± 0.05

**Table 2:** Means and their standard deviations for the average number of lambs born per lambing and the number of lambs per lifetime, as well as the heritabilities (h^2^) for each trait with their standard errors.

Breed	BD	Average Number of Lambs Born per Lambing		Number of Lambs per Lifetime	
		X ± SD	h^2^	X ± SD	h^2^
BDC	EXO	1.57 ± 0.45	0.70 ± ne	1.15 ± 0.43	0.53 ± ne
BLS	CON	1.48 ± 0.42	0.13 ± 0.04	1.03 ± 0.40	0.13 ± 0.04
CHA	MEA	1.70 ± 0.48	0.23 ± 0.05	1.18 ± 0.45	0.36 ± 0.06
COF	CON	1.50 ± 0.45	0.21 ± 0.05	1.07 ± 0.42	0.23 ± 0.15
DOS	MEA	1.45 ± 0.41	0.15 ± 0.05	1.19 ± 0.44	0.18 ± 0.04
GGH	HEA	1.26 ± 0.36	0.10 ± 0.03	0.86 ± 0.32	0.13 ± 0.04
IDF	MEA	1.62 ± 0.44	0.07 ± 0.01	1.11 ± 0.43	0.05 ± 0.01
LES	CON	1.53 ± 0.43	0.12 ± 0.02	1.09 ± 0.41	0.16 ± 0.02
MFS	MER	1.47 ± 0.41	0.06 ± 0.02	1.01 ± 0.37	0.10 ± 0.02
MLS	MER	1.60 ± 0.43	0.07 ± 0.02	1.13 ± 0.41	0.16 ± 0.02
MLW	MER	1.38 ± 0.39	0.14 ± 0.33	0.97 ± 0.33	0.09 ± 0.56
OMS	MIL	1.85 ± 0.56	0.21 ± 0.06	1.52 ± 0.59	0.24 ± 0.06
RPL	CON	1.44 ± 0.41	0.10 ± 0.03	0.93 ± 0.34	0.15 ± 0.04
SKF	MEA	1.57 ± 0.44	0.07 ± 0.01	1.14 ± 0.42	0.06 ± 0.01
SKU	HEA	1.36 ± 0.41	0.29 ± 0.07	0.86 ± 0.34	0.20 ± 0.07
SUF	MEA	1.66 ± 0.45	0.12 ± 0.03	1.18 ± 0.45	0.12 ± 0.02
SWS	MEA	2.29 ± 0.62	0.06 ± 0.09	1.89 ± 0.63	0.02 ± 0.04
TEX	MEA	1.75 ± 0.46	0.25 ± 0.05	1.36 ± 0.48	0.21 ± 0.05
WAD	CON	1.32 ± 0.42	0.25 ± 0.38	0.89 ± 0.38	0.32 ± 0.39
WGH	HEA	1.25 ± 0.37	0.30 ± 0.02	0.81 ± 0.31	0.22 ± 0.02
WHH	HEA	1.22 ± 0.32	0.07 ± 0.02	0.83 ± 0.29	0.12 ± 0.03
WKF	MEA	1.63 ± 0.44	0.12 ± 0.05	1.26 ± 0.46	0.16 ± 0.05

ne: not estimable.

**Table 3:** Heritabilities (h^2^) and their standard errors for the sheep breeds studied, with their breeding directions (BD), for the age at the first lambing in days, the lifetime from birth to fifth lambing in years and the lifetime production from first to fifth lambing.

Breed	BD	Age at First Lambing (Days)		Lifetime (Years)		Productive Live (Years)	
		X ± SD	h^2^	X ± SD	h^2^	X ± SD	h^2^
BDC	EXO	676.32 ± 206.29	0.51 ± 0.04	3.55 ± 1.48	0.10 ± 0.07	1.70 ± 1.46	0.42 ± 0.07
BLS	CON	765.19 ± 214.82	0.25 ± 0.04	4.23 ± 1.51	0.05 ± 0.03	2.14 ± 1.56	0.25 ± 0.04
CHA	MEA	706.66 ± 185.28	0.53 ± 0.05	3.30 ± 1.39	0.97 ± 0.01	1.36 ± 1.29	0.50 ± 0.02
COF	CON	735.81 ± 207.17	0.99 ± 0.01	3.78 ± 1.48	0.10 ± 0.03	1.76 ± 1.45	0.78 ± 0.03
DOS	MEA	628.56 ± 205.19	0.21 ± 0.02	3.40 ± 1.45	0.07 ± 0.03	1.68 ± 1.37	0.09 ± 0.02
GGH	HEA	774.63 ± 210.56	0.27 ± 0.02	4.00 ± 1.61	0.07 ± 0.01	1.88 ± 1.54	0.27 ± 0.02
IDF	MEA	782.36 ± 180.42	0.35 ± 0.05	4.05 ± 1.52	0.03 ± 0.04	1.91 ± 1.55	0.31 ± 0.07
LES	CON	736.17 ± 210.25	0.30 ± 0.04	4.02 ± 1.51	0.12 ± 0.03	2.00 ± 1.51	0.28 ± 0.04
MFS	MER	774.79 ± 196.34	0.08 ± 0.02	4.23 ± 1.47	0.12 ± 0.04	2.11 ± 1.49	0.07 ± 0.02
MLS	MER	781.94 ± 199.19	0.23 ± 0.03	3.98 ± 1.47	0.11 ± 0.02	1.84 ± 1.44	0.20 ± 0.02
MLW	MER	761.15 ± 171.67	0.30 ± 0.01	4.02 ± 1.45	0.02 ± 0.01	1.93 ± 1.43	0.20 ± 0.02
OMS	MIL	587.54 ± 261.08	0.21 ± 0.05	3.29 ± 1.51	0.10 ± 0.04	1.68 ± 1.43	0.22 ± 0.05
RPL	CON	798.66 ± 166.62	0.25 ± 0.04	3.98 ± 1.51	0.06 ± 0.01	1.80 ± 1.53	0.24 ± 0.02
SKF	MEA	716.31 ± 228.77	0.27 ± 0.02	3.93 ± 1.53	0.05 ± 0.01	1.97 ± 1.50	0.17 ± 0.01
SKU	HEA	786.87 ± 219.99	0.42 ± 0.02	3.55 ± 1.46	0.52 ± 0.10	1.39 ± 1.43	0.45 ± 0.02
SUF	MEA	714.87 ± 226.08	0.42 ± 0.02	3.64 ± 1.50	0.07 ± 0.01	1.68 ± 1.47	0.33 ± 0.02
SWS	MEA	582.54 ± 251.70	0.69 ± 0.02	3.42 ± 1.51	0.55 ± 0.13	1.83 ± 1.45	0.51 ± 0.06
TEX	MEA	628.02 ± 244.31	0.34 ± 0.04	3.34 ± 1.44	0.09 ± 0.03	1.62 ± 1.38	0.32 ± 0.04
WAD	CON	761.35 ± 239.12	0.15 ± 0.05	3.70 ± 1.64	0.12 ± 0.29	1.61 ± 1.56	0.13 ± 0.08
WGH	HEA	812.61 ± 181.64	0.33 ± 0.04	4.03 ± 1.48	0.25 ± 0.05	1.81 ± 1.50	0.35 ± 0.04
WHH	HEA	781.12 ± 181.41	0.34 ± 0.04	4.10 ± 1.46	0.10 ± 0.03	1.96 ± 1.44	0.33 ± 0.04
WKF	MEA	642.38 ± 236.02	0.30 ± 0.07	3.57 ± 1.49	0.08 ± 0.04	1.81 ± 1.45	0.26 ± 0.07

**Table 4:** Results for the estimates of the inbreeding depression as well as number of breeds with negative (Negative) or positive (Positive) or significantly negative (Significant-Neg) or positive (Significant-Pos) linear regression coefficients obtained from the individual rate of inbreeding (ΔF_i_), the ancestral inbreeding coefficient (F_a___Kal_) and new inbreeding coefficient (F_a___New_) according to Kalinowski, the inbreeding coefficient (F) and interaction between F and the ancestral inbreeding coefficient according to Ballou (F×F_a_Bal_) for number of lambings (Lambings), average number of lambs born per lambing (Lambs-S), number of lambs per lifetime (Lambs-L), age at the first lambing in days (AFL), lifetime from the birth to the fifth lambing in years (Lifetime) and productive life from the first to the fifth lambing in years (Prod-Life) including the corresponding standard deviations (SD), the standard errors (SE), the 95% and 5% confidence intervals (95% CI, 5% CI) and the *p*-values. Significant *p*-values are in bold.

Model			Lambings	Lambs-S	Lambs-L	AFL	Lifetime	Prod-Life
3	ΔF_i_	Mean	−1.2611	−0.7034	−0.7366	110.0823	−0.5492	−0.8732
		SD	3.0852	1.3570	1.2193	298.2234	3.1226	3.2665
		SE	0.6578	0.2893	0.2600	63.5814	0.6657	0.6964
		95% CI	2.8921	0.7829	0.2826	498.7720	3.3932	3.0218
		5% CI	−6.9471	−3.0105	−3.4292	−268.2170	−6.1832	−7.0527
		*p*-Value	0.0689	**0.0241**	**0.0100**	0.0981	0.4187	0.2237
		Negative	14	16	16	15 *	10	12
		Significant-Neg	8	5	7	1 *	9	8
4	F_a___Kal_	Mean	−436.0371	−1.1107	29.5580	11,967.4650	−478.4936	−510.2005
		SD	2035.9543	4.9456	141.0544	55,067.9862	2237.3345	2386.1934
		SE	434.0669	1.4277	30.0729	11,740.5341	477.0013	508.7381
		95% CI	8.0744	9.6708	2.4767	1716.6700	5.4092	6.2362
		5% CI	−20.0704	−9.4966	−3.7746	−413.3500	−22.3092	−11.9776
		*p*-Value	0.3266	0.4530	0.3369	0.3196	0.3272	0.3273
		Positive	8	12	11	9 #	11	8
		Significant-Pos	3	1	2	0 #	5	4
4	F_a___New_	Mean	−0.3453	−0.2796	−0.1978	17.6650	−0.0329	−0.0812
		SD	1.3962	1.5670	0.4797	134.6415	1.2222	1.2097
		SE	0.2977	0.4524	0.1023	28.7057	0.2606	0.2579
		95% CI	2.0195	3.7482	0.4060	192.5400	2.1670	1.8859
		5% CI	−2.7121	−2.3767	−0.9116	−168.4300	−1.7157	−2.2517
		*p*-Value	0.2591	0.5491	0.0667	0.5449	0.9008	0.7560
		Negative	14	15	18	15 *	10	11
		Significant-Neg	9	3	4	0 *	8	6
5	F	Mean	−0.3559	−0.2500	−0.1944	35.8330	−0.1741	−0.2739
		SD	1.5992	0.5229	0.4716	173.6980	1.5639	1.5360
		SE	0.3409	0.1115	0.1006	37.0325	0.3334	0.3275
		95% CI	1.9993	0.1845	0.3942	189.747	2.1441	1.8763
		5% CI	−2.4353	−0.6433	−0.6815	−183.165	−1.9869	−2.2458
		*p*-Value	0.3084	**0.0358**	0.0668	0.3441	0.6071	0.4123
		Negative	15	18	17	13 #	11	13
		Significant-Neg	8	0	0	2 #	7	6
5	F×F_a_Bal_	Mean	−6.3457	−136.5642	−74.0136	8345.7113	−16.7743	−63.8841
		SD	20.9937	645.3285	341.9526	38,551.9895	68.5863	293.7080
		SE	4.4759	137.5844	72.9045	8219.3118	14.6227	62.6188
		95% CI	19.1897	5.6406	4.0789	3001.639	20.0970	22.8642
		5% CI	−41.4774	−4.3203	−8.8225	−2158.215	−51.0773	−29.0590
		*p*-Value	0.1709	0.3322	0.3216	0.3215	0.2642	0.3192
		Positive	9	13	8	8 #	10	8
		Significant-Pos	6	1	1	0 #	8	6

* prolongation of AFL, # reduced AFL.

**Table 5:** Estimates of the individual rate of inbreeding (∆F_i_) for the traits number of lambings (Lambings), average number of lambs born per lambing (Lambs-S), number of lambs per lifetime (Lambs-L), age at the first lambing in days (AFL), lifetime from the birth to the fifth lambing in years (Lifetime) and productive life from the first to the fifth lambing in years (Prod-Life) and their standard errors (SE). Negative regression coefficients with *p*-values < 0.05 are in bold, except for AFL positive significant regression coefficients.

Breed	Lambings	Lambs-S	Lambs-L	AFL	Lifetime	Prod-Life
BDC	−0.7465	1.8849	1.8787	−679.5600	0.1805	1.0432
BLS	2.9471 ***	−0.5657	0.2282	384.3380	3.6016 ***	2.0333 *
CHA	−4.4996 ***	−0.3070	−1.1230 *	18.9490	−3.6462 ***	−3.4276 ***
COF	−0.6894	0.0537	−0.8051	−71.3070	0.8979 *	2.3251 *
DOS	−0.4082	0.7829	0.2241	538.2975 *	2.0771 ***	0.7465
GGH	−3.6408 ***	−0.0674	−0.5792	498.7720	−3.0190 ***	−4.4016 ***
IDF	−7.6624 ***	0.7184	−0.2884	401.9330	−6.5490 ***	−7.1328 ***
LES	−3.2224 ***	−1.1078	−1.1402	336.3790	−2.8615 ***	−3.8332 ***
MFS	0.2267	−0.3176	−0.3630	338.3338	0.6300 *	−0.2809
MLS	0.5979	−1.5811 *	−1.7305 **	379.9047	3.3932 ***	2.4438 **
MLW	−1.6796	−2.1090 *	−1.6840 *	−268.2170	−1.6587 *	−1.7940
OMS	−5.3698 ***	−3.0105 *	−3.8005 **	−238.2760	−4.7897 ***	−3.6286 **
RPL	2.8921***	−1.0729	−0.7638	−104.8430	3.2266 ***	3.6702 ***
SKF	−0.4082	0.7829	0.2241	141.5197	0.2080	−0.5240
SKU	−3.1004 ***	−1.7810 *	−1.0730 *	417.4400	−2.7385 ***	−3.8772 ***
SUF	−1.5211 ***	−0.8718	−0.9068 *	177.2593	−0.9065 ***	−1.5666 **
SWS	2.2127 **	−4.0296 *	−3.4292 *	−163.6270	2.7358 ***	3.0218 **
TEX	2.2615 ***	−0.4911	−0.2563	−67.8752	2.5810 ***	2.8208 ***
WAD	−2.2639 **	0.2025	0.0441	135.3930	−2.3835 ***	−2.7544 **
WGH	0.8952	−0.0847	0.2826	44.2000	1.0721	0.9788
WHH	−6.9471	−0.3151	−0.9744	170.8258	−6.1832	−7.0527
WKF	2.3822	−2.1886	−0.1688	31.9700	2.0495	1.9796

*: *p*-Values < 0.05, **: p-Values < 0.01, ***: p-Values < 0.001.

**Table 6:** Estimates, calculated as means across breeds, for the inbreeding depression in percentages obtained from the individual rate of inbreeding (ΔF_i_) and standardized to the respective trait mean (ΔF_i_/mean) and phenotypic (ΔF_i_/σ_P_) and additive genetic standard deviation (ΔF_i_/σ_A_) and presented as a 1% increase in ΔF_i_ for number of lambings (Lambings), average number of lambs born per lambing (Lambs-S), number of lambs per lifetime (Lambs-L), age at the first lambing in days (AFL), lifetime from the birth to the fifth lambing in years (Lifetime) and productive life from the first to the fifth lambing in years (Prod-Life), including corresponding standard deviations (SD), standard errors (SE), 95% and 5% confidence intervals (95% CI, 5% CI), skewness, kurtosis and *p*-values. Significant *p*-values are in bold.

Model			Lambings	Lambs-S	Lambs-L	AFL	Lifetime	Prod-Life
3	ΔF_i_/mean	Mean	−0.4762	−0.4142	−0.6058	0.1372	−0.1456	−0.5126
		Median	−0.2672	−0.2696	−0.7151	0.1877	−0.0519	−0.1998
		SD	1.1234	0.7781	0.9058	0.4236	0.8247	1.8283
		SE	0.2395	0.1659	0.1931	0.0903	0.1758	0.3898
		95% CI	0.9730	0.5393	0.3489	0.6439	0.8510	1.7445
		5% CI	−2.4083	−1.6280	−1.8141	−0.4056	−1.5095	−3.6049
		Skewness	−0.4163	−0.0574	0.2020	−0.5776	−0.3918	−1.2420
		Kurtosis	−0.7747	−0.4592	0.7823	0.0529	−1.1015	−0.3109
		*p*-Value	0.0600	**0.0209**	**0.0050**	0.1435	0.4171	0.2027
3	ΔF_i_/σ_P_	Mean	−4.1006	−1.7037	−2.2870	0.5797	−7.4619	−2.0265
		Median	−1.8584	−1.1294	−2.5742	0.6198	−0.7392	−0.8084
		SD	11.0909	3.0268	3.3149	1.9720	33.2084	8.6519
		SE	2.3646	1.9130	0.7067	0.4204	1.0837	1.8446
		95% CI	11.8565	2.0378	1.1250	2.9316	25.6819	11.1385
		5% CI	−21.1382	−6.2475	−7.3648	−2.0280	−48.8846	−17.0021
		Skewness	−0.4591	−0.1528	0.0644	−0.5776	−1.60146	−0.2747
		Kurtosis	−0.4503	−0.5337	0.6009	0.0529	3.70292	−0.7410
		*p*-Value	0.0975	**0.0153**	**0.0040**	0.1824	0.3039	0.2844
3	ΔF_i_/σ_A_	Mean	−12.9856	−5.3126	−7.8731	1.3670	−23.5479	−3.8229
		Median	−9.1105	−3.7926	−5.7905	1.1907	−2.9698	−1.7192
		SD	34.9760	9.2699	13.1590	3.7161	113.4436	15.6520
		SE	7.4569	1.9764	2.8055	0.7923	24.1863	3.3370
		95% CI	26.9690	6.6416	2.9683	7.6627	113.8926	15.5837
		5% CI	−77.6709	−16.1786	−20.8874	−4.2086	−207.0976	−30.1416
		Skewness	−0.5662	−1.0357	−2.6339	−0.0587	−1.3802	−0.8095
		Kurtosis	0.2060	1.7642	9.3215	−0.6017	2.6077	7.3876
		*p*-Value	0.0962	**0.0138**	**0.0106**	0.0991	0.3413	0.2649

## Data Availability

Restrictions apply to the availability of these data. Data were obtained from vit/Verden and are on a reasonable request available from the authors with the permission of the German sheep breeding associations.

## Supplementary Materials

The following supporting information can be downloaded at: https://www.mdpi.com/article/10.3390/ani14223214/s1, Table S1. Survey on the data used for the present analysis showing the number and percentages of ewes per birth year (*n* = 62,198 ewes) and birth month, the distribution of the first lambing years and all lambing years (*n* = 173,495). Table S2. Number of animals per breed and phenotypic data with minimum, maximum, average and standard deviation for the different fertility and longevity traits. Table S3. Genetic parameters for the traits the number of lambings, the average number of lambs born per lambing and the number of lambs per lifetime. Table S4. Genetic parameters for the traits age at the first lambing in days, length of lifetime from the birth to the fifth lambing in years and the length productive life from the first to the fifth lambing in years. Table S5a. Estimates of the individual rate of inbreeding (ΔFi) on the number of lambings and their corresponding standard errors (SE) and *p*-values by breed. Table S5b. Estimates of the ancestral (F_a_Kal_) and new (F_a_New_) inbreeding coefficients according to Kalinowski on the number of lambings and their corresponding standard errors (SE) and *p*-values by breed. Table S5c. Estimates of the inbreeding coefficient (F) and interaction between F and the ancestral inbreeding coefficient according to Ballou (F×F_a_Bal_) on the number of lambings and their corresponding standard errors (SE) and *p*-values by breed. Table S5d. Estimates of the inbreeding depression, inferred from the individual rate of inbreeding (ΔF_i_) and the ancestral (F_a___Kal_) and new (F_a___New_) inbreeding coefficients according to Kalinowski, inbreeding (F) and interaction between F and the ancestral inbreeding coefficient according to Ballou (F×F_a_Bal_) on the number of lambings and their corresponding standard deviations (SD), standard errors (SE), 95% and 5% confidence intervals (95% CI, 5% CI), and *p*-Values for all breeds, as well as the four breeding directions (BDs) including merino (MER), meat (MEA), country (CON), and heath (HEA). Table S6a. Estimates of the individual rate of inbreeding (ΔFi) on the average number of lambs born per lambing and their corresponding standard errors (SE) and *p*-values by breed. Table S6b. Estimates of the ancestral (F_a_Kal_) and new (F_a_New_) inbreeding coefficients according to Kalinowski on the average number of lambs born per lambing and their corresponding standard errors (SE) and *p*-values by breed. Table S6c. Estimates of the inbreeding coefficient (F) and interaction between F and the ancestral inbreeding coefficient according to Ballou (F×F_a_Bal_) on the average number of lambs born per lambing and their corresponding standard errors (SE) and *p*-values by breed. Table S6d. Estimates of the inbreeding depression, inferred from the individual rate of inbreeding (ΔF_i_) and the ancestral (F_a___Kal_) and new (F_a___New_) inbreeding coefficients according to Kalinowski, inbreeding (F) and interaction between F and the ancestral inbreeding coefficient according to Ballou (F×F_a_Bal_) on the average number of lambs born per lambing and their corresponding standard deviations (SD), standard errors (SE), 95% and 5% confidence intervals (95% CI, 5% CI), and *p*-values for all breeds, as well as the four breeding directions (BDs) including merino (MER), meat (MEA), country (CON), and heath (HEA). Table S7a. Estimates of the individual rate of inbreeding (ΔFi) on the number of lambs per life and their corresponding standard errors (SE) and *p*-values by breed. Table S7b. Estimates of the ancestral (F_a_Kal_) and new (F_a_New_) inbreeding coefficients according to Kalinowski on the number of lambs per life and their corresponding standard errors (SE) and *p*-values by breed. Table S7c. Estimates of the inbreeding coefficient (F) and interaction between F and the ancestral inbreeding coefficient according to Ballou (F×F_a_Bal_) on the number of lambs per life and their corresponding standard errors (SE) and *p*-values by breed. Table S7d. Estimates of the inbreeding depression, inferred from the individual rate of inbreeding (ΔF_i_) and the ancestral (F_a___Kal_) and new (F_a___New_) inbreeding coefficients according to Kalinowski, inbreeding (F) and interaction between F and the ancestral inbreeding coefficient according to Ballou (F×F_a_Bal_) on the number of lambs per life and their corresponding standard deviations (SD), standard errors (SE), 95% and 5% confidence intervals (95% CI, 5% CI), and *p*-values for all breeds, as well as the four breeding directions (BDs) including merino (MER), meat (MEA), country (CON), and heath (HEA). Table S8a. Estimates of the individual rate of inbreeding (ΔFi) on the age at first lambing in days and their corresponding standard errors (SE) and *p*-values by breed. Table S8b. Estimates of the ancestral (F_a_Kal_) and new (F_a_New_) inbreeding coefficients according to Kalinowski on the age at first lambing in days and their corresponding standard errors (SE) and *p*-values by breed. Table S8c. Estimates of the inbreeding coefficient (F) and interaction between F and the ancestral inbreeding coefficient according to Ballou (F×F_a_Bal_) on the age at first lambing in days and their corresponding standard errors (SE) and *p*-values by breed. Table S8d. Estimates of the inbreeding depression, inferred from the individual rate of inbreeding (ΔF_i_), and the ancestral (F_a___Kal_) and new (F_a___New_) inbreeding coefficients according to Kalinowski, inbreeding (F) and interaction between F and the ancestral inbreeding coefficient according to Ballou (F×F_a_Bal_) on the age at first lambing in days and their corresponding standard deviations (SD), standard errors (SE), 95% and 5% confidence intervals (95% CI, 5% CI), and *p*-values for all breeds, as well as the four breeding directions (BDs) including merino (MER), meat (MEA), country (CON), and heath (HEA). Table S9a. Estimates of the individual rate of inbreeding (ΔFi) on the lifetime from birth to fifth lambing and their corresponding standard errors (SE) and *p*-Values by breed. Table S9b. Estimates of the ancestral (F_a_Kal_) and new (_Fa_New_) inbreeding coefficients according to Kalinowski on the lifetime from birth to fifth lambing and their corresponding standard errors (SE) and *p*-values by breed. Table S9c. Estimates of the inbreeding coefficient (F) and interaction between F and the ancestral inbreeding coefficient according to Ballou (F×F_a_Bal_) on the lifetime from birth to fifth lambing and their corresponding standard errors (SE) and *p*-values by breed. Table S9d. Estimates of the inbreeding depression, inferred from the individual rate of inbreeding (ΔF_i_), the ancestral (F_a___Kal_) and new (F_a___New_) inbreeding coefficients according to Kalinowski, inbreeding (F) and interaction between F and the ancestral inbreeding coefficient according to Ballou (F×F_a_Bal_) on the life time from birth to fifth lambing and their corresponding standard deviations (SD), standard errors (SE), 95% and 5% confidence intervals (95% CI, 5% CI), and *p*-values for all breeds, as well as the four breeding directions (BDs) including merino (MER), meat (MEA), country (CON), and heath (HEA). Table S10a. Estimates of the individual rate of inbreeding (ΔFi) on the productive life from first to fifth lambing and their corresponding standard errors (SE) and *p*-values by breed. Table S10b. Estimates of the ancestral (F_a_Kal_) and new (F_a_New_) inbreeding coefficients according to Kalinowski on the productive life from first to fifth lambing and their corresponding standard errors (SE) and *p*-values by breed. Table S10c. Estimates of the inbreeding coefficient (F) and interaction between F and the ancestral inbreeding coefficient according to Ballou (F×F_a_Bal_) on the productive life from first to fifth lambing and their corresponding standard errors (SE) and *p*-values by breed. Table S10d. Estimates of the inbreeding depression, inferred from the individual rate of inbreeding (ΔF_i_) and the ancestral (F_a___Kal_) and new (F_a___New_) inbreeding coefficients according to Kalinowski, inbreeding (F) and interaction between F and the ancestral inbreeding coefficient according to Ballou (F×F_a_Bal_) on the productive life from birth to fifth lambing and their corresponding standard deviations (SD), standard errors (SE), 95% and 5% confidence intervals (95% CI, 5% CI), and *p*-Values for all breeds, as well as the four breeding directions (BDs) including merino (MER), meat (MEA), country (CON), and heath (HEA). Table S11. Estimates, calculated as means across breeds, of the inbreeding depression in percentages, inferred from the inbreeding coefficient (F) and calibrated to the respective trait mean (F/mean), phenotypic (F/σ_P_) and additive genetic standard deviation (F/σ_A_) and presented as a 1% increase in F for number of lambings (Lambings), average number of lambs born per lambing (Lambs-S), number of lambs per lifetime (Lambs-L), age at the first lambing in days (AFL), lifetime from birth to fifth lambing in years (Lifetime) and productive life from the first to the fifth lambing (Prod-Life) and their corresponding standard deviations (SD), standard errors (SE), 95% and 5% confidence intervals (95% CI, 5% CI), skewness, kurtosis and *p*-values. Significant *p*-values are in bold.
